# Effects of a 12-Week Supervised Exercise Program on Quality of Life, Functional Capacity, and Biological Parameters in Women with Breast Cancer: A Prospective Pilot Program

**DOI:** 10.3390/jcm15041480

**Published:** 2026-02-13

**Authors:** Gema Barrientos Vicho, Luis Posado-Dominguez, José David Urchaga, Luis Figuero-Perez, Francisco Javier Alves Vas, Belén Cigarral García, Iñaki Pérez Echepare, Rebeca Lozano Mejorada, Joaquín Martín Pena, Monserrat Diaz Martín, Yolanda López-Mateos, Maria Garijo-Martínez, Juan Carlos Redondo-González, Jonathan Roldán-Ruiz, Emilio Fonseca Sánchez, Cesar Augusto Rodríguez Sánchez

**Affiliations:** 1 Faculty of Education, Physical Activity and Sport Sciences, Pontifical University of Salamanca, 37002 Salamanca, Spain; fjalvesva@upsa.es (F.J.A.V.);; 2 Medical Oncology Service, University Hospital of Salamanca, 37007 Salamanca, Spain; lfiguero@saludcastillayleon.es (L.F.-P.); jcredondo@saludcastillayleon.es (J.C.R.-G.); jroldanr@saludcastillayleon.es (J.R.-R.);; 3 Institute of Biomedical Research of Salamanca, 37007 Salamanca, Spain; 4 Faculty of Communication, Pontifical University of Salamanca, 37002 Salamanca, Spain; jdurchagali@upsa.es; 5 Medical Oncology Service, Virgen de la Concha Hospital, 49022 Zamora, Spain; belencigarral@saludcastillayleon.es; 6 Faculty of Medicine, University of Salamanca, 37008 Salamanca, Spain

**Keywords:** breast cancer, supervised exercise, quality of life, metastatic breast cancer, functional capacity, supportive care, oncology rehabilitation

## Abstract

**Objective**: Physical exercise is an effective supportive strategy in oncology, yet its implementation remains limited, particularly in patients with metastatic breast cancer. This prospective pilot program aimed to evaluate the effects of a 12-week supervised multicomponent exercise program on quality of life, functional capacity, and biological parameters in women undergoing treatment for breast cancer. **Methods**: Prospective single-arm pilot program. In total, 44 women with early or metastatic breast cancer were enrolled; 29 completed the full intervention and assessments. The program consisted of supervised aerobic, resistance, mobility, and neuromuscular training three times per week. Quality of life was assessed using the EORTC QLQ-C30 questionnaire. Strength, body composition, functional mobility (Functional Movement Screen, FMS), 6 min walk test (6MWT), and laboratory markers (RDW, HDL, and cholesterol profile) were evaluated pre- and post-intervention. **Results**: Significant improvements were observed in global quality of life (+19.5%, *p* = 0.002), physical function (*p* = 0.006), emotional function (*p* = 0.003), and fatigue reduction (*p* = 0.007). The FMS total score increased significantly (*p* = 0.001), and 6MWT distance improved by 110 m (*p* < 0.001). Biochemical analyses showed a significant reduction in RDW (*p* = 0.005), a pro-inflammatory marker, and an increase in HDL cholesterol (*p* = 0.007). The intervention was well tolerated, with no exercise-related serious adverse events and high adherence to the supervised program. **Conclusions**: A 12-week supervised exercise program is feasible and beneficial for women with breast cancer, including those with metastatic disease. It is associated with improvements in quality of life, functional mobility, and selected markers of systemic inflammation. These findings should be considered exploratory and support the integration of structured exercise into routine oncologic care, pending confirmation in larger controlled studies.

## 1. Introduction

Breast cancer represents a major public health challenge worldwide due to its high incidence and prevalence and the significant morbidity and mortality associated with the disease [1]. In Spain, estimates from the Spanish Society of Medical Oncology indicated that 296,103 new cancer cases were expected to be diagnosed in 2025, representing a 3.3% increase compared to the previous year. In 2022, breast cancer was the second most commonly diagnosed tumor in both sexes and the fourth leading cause of cancer-related mortality, with 34,735 new cases and 6747 deaths [2].

As more patients live longer with the disease—including those with metastatic involvement—the focus of care increasingly extends beyond oncologic control to preserving function, alleviating treatment-related symptoms, and maintaining quality of life. Many women experience fatigue, muscle and bone loss, mood disturbances, and cardiorespiratory limitations during treatment, which may worsen toward the end of life and negatively affect daily activities and independence [3,4].

In this evolving clinical scenario, patients with metastatic breast cancer remain underrepresented in exercise oncology research, as many intervention studies have focused on early-stage disease or survivorship settings, frequently excluding individuals undergoing active systemic treatment due to safety or feasibility concerns. Consequently, evidence on the feasibility and potential benefits of supervised exercise programs during active treatment in women with metastatic breast cancer remains limited.

Supervised physical exercise has emerged as a promising supportive strategy capable of mitigating some treatment-related toxicities, improving functional capacity, and enhancing overall well-being. However, despite growing evidence supporting its benefits, implementation in routine clinical practice remains limited due to structural barriers, lack of standardized programs, and insufficient integration across care pathways [5,6].

Multidisciplinary collaboration—including oncology providers, rehabilitation specialists, psychology, physiotherapy, and professionals in exercise science—is critical to enable safe, individualized exercise prescription. Nevertheless, further research is needed to strengthen the evidence base, particularly during active treatment, and clarify the impact of structured exercise on functional, metabolic, and patient-reported outcomes.

Therefore, the objective of this prospective pilot study was to evaluate the potential effects of a 12-week supervised multicomponent exercise program on health-related quality of life (QoL) and physical function in women undergoing treatment for breast cancer.

## 2. Material and Methods

This prospective study was conducted as part of an interdisciplinary collaboration between specialists in Physical Activity and Sports Sciences at the Pontifical University of Salamanca and a team of Medical Oncologists specializing in breast cancer at the University Hospital of Salamanca. The study aimed to evaluate the effects of a 12-week supervised exercise program on patients undergoing adjuvant, neoadjuvant, or metastatic treatment for breast cancer.

### 2.1. Primary and Secondary Objetives

The objective of this prospective pilot study was to evaluate the impact of a 12-week supervised exercise program on health-related QoL in women undergoing treatment for breast cancer.

Secondary endpoints included muscular strength, body composition, functional capacity (6MWT (Six-Minute Walk Test) and Functional Movement Screen (FMS)), and hematological and lipid biomarkers. We also assessed feasibility, adherence and adverse events.

### 2.2. Recruitment, Follow-Up and Variables of the Study

Patients were eligible for inclusion if they met the following criteria: women under 65 years of age, an Eastern Cooperative Oncology Group (ECOG) performance status of 0–1, as assessed by the treating medical oncologist during routine clinical evaluation, and active oncologic treatment (adjuvant, neoadjuvant, or for metastatic disease). Patients with bone metastases were eligible provided they had no unstable or symptomatic skeletal lesions and were clinically cleared by their treating oncologist to participate in supervised exercise. Recruitment was conducted during routine medical oncology consultations, where a total of 50 women were invited to participate. Of these, 44 agreed to enroll in the study. Prior to starting the program, participants underwent a baseline assessment that included:

- Laboratory analyses: Hemoglobin, red cell distribution width (RDW), total cholesterol, high-density lipoprotein (HDL) cholesterol, low-density lipoprotein (LDL) cholesterol, and triglycerides.
- Strength assessments, detailed in File S1.
- QoL using the EORTC QLQ-C30 questionnaire, including multiple subdomains (Supplementary File S2).

### 2.3. Exercise Intervention

Each session consisted of a structured, multicomponent routine including a warm-up (5–10 min of mobility and dynamic stretching), a main aerobic phase (20–30 min of continuous or interval training at moderate intensity, rating of perceived exertion (RPE) 12–15, 50–85% hearth rate reserve (HRR)), resistance training targeting major muscle groups (2–4 sets, 8–12 repetitions at 30–60% estimated 1 repetition maximum (RM)), and a cool-down with stretching. Training volume and intensity progressed every 2–3 weeks and were individually adjusted based on tolerance, treatment phase, and clinical limitations. All exercise sessions were supervised by professionals in Exercise Science. Session attendance was recorded at each training session by the supervising exercise professionals. Adherence was defined as the proportion of scheduled exercise sessions attended by each participant over the 12-week intervention period. A detailed session-by-session plan is provided in Supplementary File S3.

### 2.4. Statistical Analyses

Statistical analyses were performed using SPSS v28 and RStudio Desktop version 2023.09.1+494. A descriptive analysis of baseline characteristics was conducted, reporting means and standard deviations for continuous variables and frequencies for categorical variables.

Data distribution was assessed for normality using the Shapiro–Wilk test. To compare pre- and post-intervention measurements, different statistical tests were applied according to data distribution: the paired Student’s *t*-test for normally distributed continuous variables and the Wilcoxon signed-rank test for non-normally distributed continuous variables.

Two-tailed significance testing was conducted to evaluate changes in quality of life, strength parameters, flexibility, body composition, and laboratory values after the 12-week intervention. A *p*-value < 0.05 was considered statistically significant. Given the exploratory nature of this pilot study and the limited sample size, no formal correction for multiple comparisons was applied; therefore, results should be interpreted with caution, acknowledging an increased risk of type I error.

Given the exploratory nature of this pilot study and the limited sample size, confidence intervals and mean differences were not systematically reported for all outcomes.

Results are therefore presented descriptively to identify potential trends to be confirmed in future controlled studies.

## 3. Results

**Figure 1:**
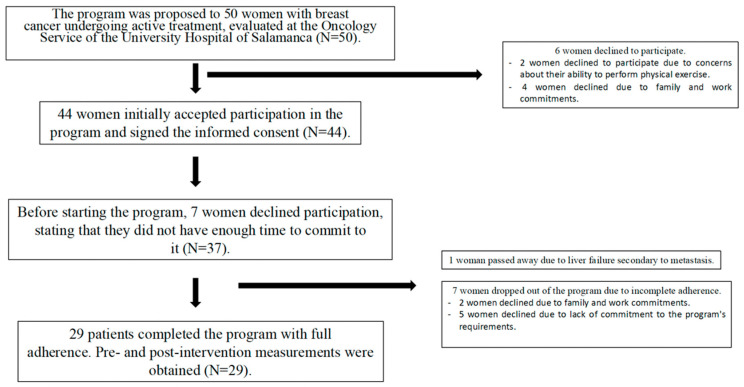
Flow of participant recruitment, enrollment, and study completion across the 12-week intervention.

During the initial phase, 7 participants withdrew from the study before the intervention began, leaving 37 active participants. Over the course of the program, one patient died due to hepatic failure secondary to tumor-related pseudocirrhosis. Additionally, 7 participants completed the intervention partially or did not undergo final evaluations (including laboratory and strength measurements). As a result, 29 participants completed the study with full pre- and post-intervention data and were included in the final analysis (Figure 1).

(1) Baseline characteristics of the sample

A total of 29 patients diagnosed with breast cancer were included, with a mean age of 50.07 ± 6.65 years. At diagnosis, 34.5% (n = 10) had metastatic disease, with the most common metastatic sites being bone (34.5%), liver (17.2%), and lung (13.8%). Regarding treatment, 27.6% (n = 8) were receiving adjuvant chemotherapy, and 34.5% (n = 10) were undergoing treatment for metastatic disease.

Sociodemographic data revealed that 27.6% (n = 8) of patients were actively employed, and 37.9% (n = 11) had a history of tobacco use. Most patients (48.3%, n = 14) had a university degree. Comorbidities included antidepressant use (20.7%, n = 6), benzodiazepine use (34.5%, n = 10), and other medications such as antihypertensives and statins.

Regarding treatment history, 93.1% (n = 27) underwent surgery, with lumpectomy being the most common procedure (58.6%, n = 17). Radiation therapy was received by 65.5% (n = 19) of the cohort.

Adverse events during the intervention were minimal, with 13.7% (n = 4) requiring emergency visits and 6.9% (n = 2) requiring hospitalization. No injuries leading to functional impairment were reported. A comprehensive summary of baseline characteristics is shown in Table 1.

(2) Results of the EORTC QLQ-C30 Quality of Life Questionnaire

QoL was assessed using the EORTC QLQ-C30 questionnaire. Global quality of life (QL2.S) increased from 58.91 (±18.22) to 70.40 (±13.83), a statistically significant change (*p* = 0.002).

Improvements were also observed in the functional scales: physical function from 85.06 to 91.95 (*p* = 0.006), role function from 74.71 to 89.66 (*p* = 0.004), emotional function from 77.01 to 86.78 (*p* = 0.003), cognitive function from 73.56 to 81.03 (*p* = 0.034), and social function from 64.94 to 78.74 (*p* = 0.005).

Among symptom scales, fatigue decreased from 37.55 to 26.82 (*p* = 0.007), while no statistically significant differences were observed in pain, nausea/vomiting, appetite loss, constipation, diarrhea, dyspnea, insomnia or financial difficulties (all *p* > 0.05).

**Figure 2:**
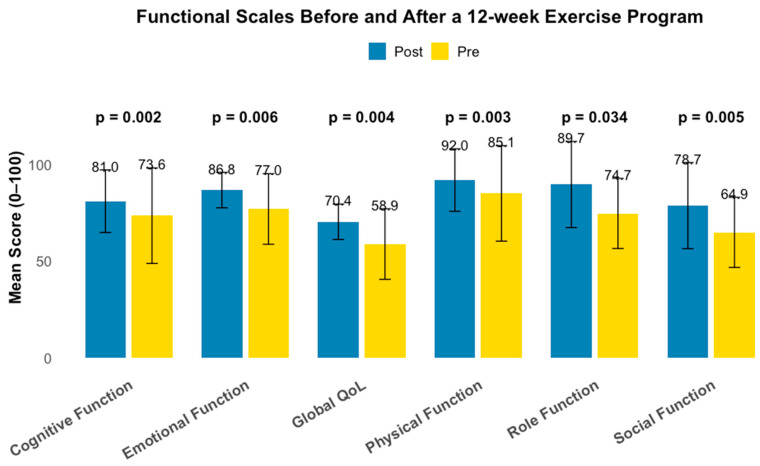
Pre-intervention (yellow bars) and post-intervention (blue bars) comparison of EORTC QLQ-C30 functional scale scores after a 12-week supervised exercise program. Bars represent mean values, and error bars indicate standard deviations. *p*-values correspond to paired pre–post comparisons.

A full summary of QLQ-C30 subscales is presented in Table 2 and Figure 2.

(3) Global Analysis of the FMS

The FMS total score increased from 2.23 (±0.46) at baseline to 2.51 (±0.31) after the 12-week intervention.

**Figure 3:**
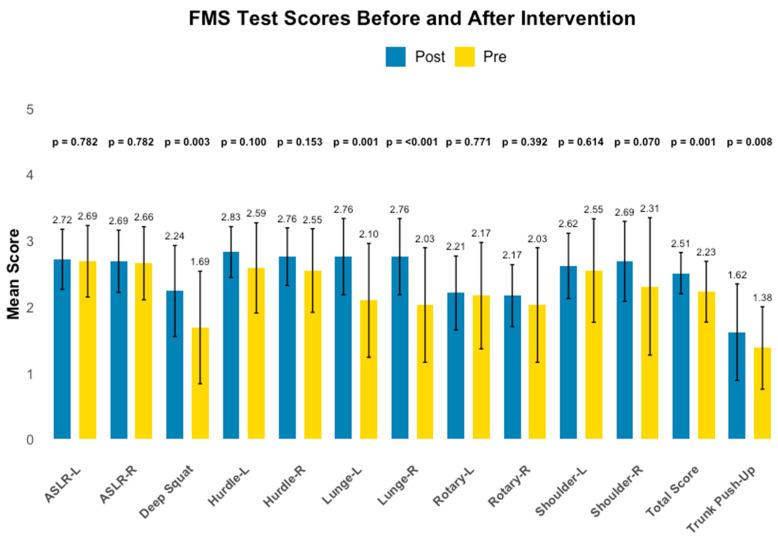
Mean scores of Functional Movement Screen (FMS) tests before (yellow bars) and after (blue bars) a 12-week supervised exercise intervention. Bars represent mean values, and error bars indicate standard deviations. Abbreviations: ASLR, Active Straight-Leg Raise; L, Left; R, Right.

This change was statistically significant (Z = −3.218; *p* = 0.001). Individual component scores are presented in Table 3, and overall results are illustrated in Figure 3.

Deep Squat (FMS.1) increased from 1.69 (±0.85) to 2.24 (±0.68) (Z = −3.017; *p* = 0.003). Inline Lunge improved on both sides: right side from 2.03 (±0.86) to 2.76 (±0.57) (Z = −3.384; *p* < 0.001) and left side from 2.10 (±0.86) to 2.76 (±0.57) (Z = −3.275; *p* = 0.001). Trunk Stability Push-Up increased from 1.38 (±0.62) to 1.62 (±0.72) (Z = −2.646; *p* = 0.008). Other assessments, such as Shoulder Mobility (FMS.4) and Rotary Stability (FMS.7), showed non-significant changes (*p* = 0.070 and *p* = 0.392, respectively).

(4) The 6-Minute Walk Test (6MWT)

**Figure 4:**
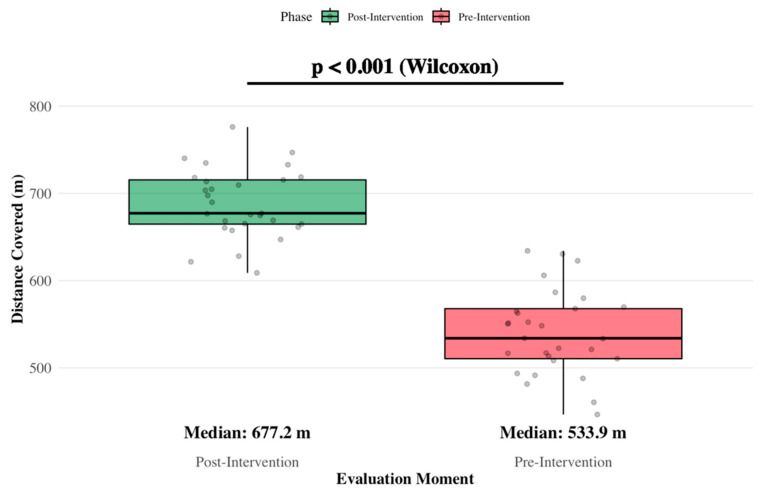
Evolution of Functional Capacity (6MWT).

The 6MWT was performed at baseline and after the 12-week intervention. The mean distance covered increased from 556.21 m (SD = 123.60) at baseline to 666.38 m (SD = 56.07) following the program, representing a mean difference of 110.17 m (19.8%). This improvement was statistically significant (Z = −4.381; *p* < 0.001) (Figure 4).

(5) Analysis of Analytical Parameters Pre- and Post-Intervention

- Hematological Parameters

Hb levels and RDW were assessed before and after the intervention. Mean hb increased from 12.80 g/dL (±1.40) to 12.97 g/dL (±1.75), although this change was not statistically significant (t = −0.954; *p* = 0.174).

**Figure 5:**
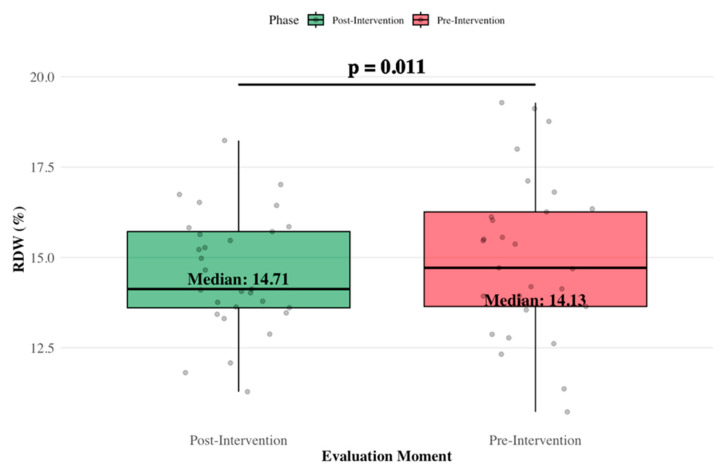
RDW levels before and after the exercise intervention in breast cancer patients.

In contrast, RDW decreased from 15.21% (±2.28) to 14.18% (±1.87), with the reduction reaching statistical significance (t = 2.435; *p* = 0.011) (Figure 5). Corresponding effect sizes for these variables are presented in Table 4.

- Metabolic Parameters (Lipid Profile)

Total cholesterol, triglycerides, HDL cholesterol, and LDL cholesterol levels were assessed before and after the 12-week intervention. Total cholesterol decreased from 195.48 mg/dL (±33.67) to 188.97 mg/dL (±34.07), although this change did not reach statistical significance (t = 1.601; *p* = 0.060).Triglycerides also showed a reduction, from 109.52 mg/dL (±55.73) to 104.07 mg/dL (±43.99), without statistical significance (t = 0.665; *p* = 0.256). HDL cholesterol increased from 60.66 mg/dL (±15.48) to 64.55 mg/dL (±16.66), a statistically significant change (t = −2.984; *p* = 0.003). Finally, while a numerical reduction in LDL cholesterol was observed, this change did not reach statistical significance (*p* = 0.172). Effect sizes for these comparisons are shown in Table 4.

## 4. Discussion

Breast cancer remains a major public health challenge worldwide, with incidence rates continuing to rise as advances in early detection and systemic therapies extend survival. Therefore, an increasing number of women are living longer with the disease, including those with metastatic breast cancer (MBC), for whom maintaining quality of life has become a central component of care [1].

While the benefits of exercise are well established in early-stage breast cancer and survivorship, the role of structured physical activity in women with MBC has been comparatively less explored, particularly during active oncologic treatment. In this context, we conducted a pilot study to examine the feasibility and potential effects of a 12-week multicomponent exercise program in women with breast cancer, including those with MBC.

Adherence is a key consideration when implementing exercise programs in patients with metastatic breast cancer, as participation continuity may influence both feasibility and the magnitude of potential benefits. In our cohort, 37 of the 50 women to whom the program was offered agreed to participate, corresponding to a recruitment rate of 74%, which compares favorably with that reported in large randomized trials such as the PREFERABLE-EFFECT study, where 357 of 856 invited patients were enrolled (recruitment rate 41.7%) [7]. Among participants who initiated the intervention, a 19% dropout rate was observed, underscoring the need to identify and address barriers that limit sustained engagement in exercise interventions.

Notably, this attrition rate is comparable to those reported in PREFERABLE-EFFECT, in which overall dropout rates reached 12.3% at 3 months, 18.5% at 6 months, and 24.1% at 9 months, despite the structured and supervised nature of the intervention [7]. Taken together, these findings suggest that recruitment and attrition rates observed in our pilot study are consistent with, and in some aspects more favorable than, those reported in large-scale trials, supporting the real-world feasibility of supervised exercise programs in women with metastatic breast cancer. Factors such as transportation challenges, financial strain, limited social support, and treatment-related symptom burden are likely contributors to discontinuation and should be systematically addressed when designing future exercise initiatives for this population.

Previous research suggests that adherence in individuals with metastatic cancer may improve through flexible and accessible models, including home-based programs, hybrid formats combining supervised and remote sessions, and personalized follow-up strategies aimed at maintaining engagement over time [7,8].

Participation in the 12-week intervention was associated with meaningful improvements in multiple domains of health-related quality of life (HRQoL). Global quality of life increased from 58.91 ± 18.22 to 70.40 ± 13.83, representing a 19.5% relative improvement. Significant gains were also observed in physical function (85.06 ± 13.76 to 91.95 ± 9.15), role function (74.71 ± 24.65 to 89.66 ± 13.67), emotional function (77.01 ± 17.35 to 86.78 ± 16.14), and social function (64.94 ± 23.71 to 78.74 ± 22.23). Fatigue scores markedly decreased from 37.55 ± 18.41 to 26.82 ± 15.29, highlighting the relevance of structured exercise in mitigating one of the most prevalent and debilitating symptoms in this population. Taken together, these multidimensional improvements suggest that supervised exercise may provide broad benefit across physical, emotional, and social domains during active treatment.

In addition to changes in patient-reported outcomes, objective measures of physical function also improved following the intervention. The distance covered in the six-minute walk test increased substantially, reflecting enhancement in cardiorespiratory fitness and submaximal exercise tolerance. Improvements were similarly observed in movement quality assessed by the Functional Movement Screen (FMS). The total score increased from 2.23 ± 0.46 to 2.51 ± 0.31 (*p* = 0.001), with the largest gains observed in the Deep Squat (*p* = 0.003), Incline Lunge (*p* < 0.001 and *p* = 0.001), and Trunk Stability Push-Up (*p* = 0.008). These changes suggest better lower-limb control, mobility, and trunk stability, which are relevant for preserving function and independence during oncologic treatment.

Beyond functional improvements, the intervention was associated with favorable trends in selected biological parameters. A significant reduction in red cell distribution width (RDW) was observed (from 15.21% to 14.18%; *p* = 0.005), alongside an increase in HDL cholesterol (from 60.66 to 64.55 mg/dL; *p* = 0.007). While these changes should be interpreted with caution, they may indicate a beneficial modulation of systemic inflammation and metabolic health [9]. In this regard, previous studies conducted in cardiovascular populations have shown that exercise training is associated with reductions in RDW and improvements in exercise tolerance, with RDW inversely correlated with peak oxygen uptake [10].

The program was well tolerated, and no exercise-related injuries or adverse events were reported, supporting the feasibility of implementing supervised exercise interventions in women receiving diverse oncologic treatments. These findings are consistent with previous studies demonstrating that supervised exercise programs are safe and feasible across different cancer populations, including patients undergoing active systemic therapy [4,5,7]. These results should be interpreted cautiously, as this was a single-arm study conducted in a clinically heterogeneous population; however, the observed improvements in global quality of life and selected exercise-sensitive domains are consistent with findings reported in randomized studies during active breast cancer treatment [11].

Importantly, participants in our cohort represented a broad spectrum of disease stages and therapeutic pathways, reflecting a real-world oncology setting. During the intervention period, one participant died due to liver failure secondary to pseudocirrhosis, a complication related to disease progression rather than the exercise program, and another participant died six months after completing the intervention as a consequence of brain metastases. These events highlight the advanced and heterogeneous nature of the study population and underscore that, even in patients approaching the end of life, appropriately tailored and supervised physical activity may still have a role, focusing on functional preservation, symptom relief, and quality of life rather than disease modification.

Several limitations must be acknowledged. The small sample size (N = 29) and lack of a control group limit the ability to attribute observed changes solely to the intervention, and the findings should therefore be interpreted as exploratory. Accordingly, causal inferences cannot be established, and the observed effects should be considered preliminary. Future studies employing randomized controlled designs will be required to confirm the effectiveness of supervised exercise interventions in this population. Variability in adherence and incomplete follow-up in some participants may also have influenced results. In addition, participant attrition may have introduced selection bias, as individuals who completed the intervention could differ from those who withdrew in terms of symptom burden, treatment-related toxicity, logistical barriers, or motivation. Consequently, the observed benefits may overestimate the effects achievable in the broader population of women undergoing breast cancer treatment, thereby limiting the generalizability of the findings. Additionally, the 12-week duration did not allow assessment of long-term maintenance of benefits or potential cumulative effects of continued training.

Despite these limitations, this study adds to the growing evidence supporting supervised exercise as a feasible, safe, and potentially beneficial component of supportive care for women undergoing treatment for breast cancer, including those with advanced disease. The multidisciplinary model developed in collaboration with trained professionals in Exercise Science from the Pontifical University of Salamanca demonstrates that exercise programs can be successfully implemented in partnership with non-oncology specialists, even within routine clinical care.

Importantly, the increasing clinical relevance of exercise in oncology is now reflected in international clinical practice guidelines. Recent ESMO recommendations for localized colon cancer have explicitly incorporated concomitant participation in structured exercise programmes as part of adjuvant management, underscoring a paradigm shift in which physical exercise is no longer considered merely a lifestyle intervention, but an evidence-based component of comprehensive oncologic care [12]. Although these recommendations currently apply to colon cancer, they reinforce the broader integration of structured exercise into routine oncology practice and provide strong external validity to supervised exercise interventions such as the program evaluated in the present study.

Future research should prioritize adequately powered randomized trials to confirm the benefits suggested here and explore longer-term outcomes beyond the intervention window. Economic evaluations will also be essential, as integrating structured exercise into public healthcare pathways may reduce treatment-related morbidity, delay functional decline, and potentially mitigate healthcare costs through decreased symptom burden, fewer complications, and reduced reliance on pharmacologic interventions. Establishing exercise as a sustainable service within oncology may represent both a clinical and a cost-effective advancement in comprehensive cancer care.

## 5. Conclusions

This pilot study suggests that a 12-week supervised exercise program is feasible, safe, and associated with improvements in quality of life and physical function in women undergoing treatment for breast cancer. Positive changes in movement quality, functional capacity, and selected biomarkers (RDW and HDL cholesterol) further support the potential value of integrating structured exercise into routine oncology care. These findings warrant confirmation in larger, controlled studies designed to evaluate long-term effects and strategies to optimize adherence.

## Figures and Tables

**Table 1:** Baseline characteristics of the sample.

Characteristic (N = 29)	**Mean (+/− SD)**
Age	50.07 ± 6.65
Age at diagnosis	46.21 ± 7.12
BMI (pre-intervention)	25.26 ± 4.98
Fat mass (pre-intervention)	44.68 ± 5.69
Waist circumference (pre-intervention)	81.69 ± 11.58 cm
Hip circumference (pre-intervention)	102.0 ± 10.97 cm
Characteristic (N = 29)	N (%)
Current oncologic treatment at baseline - Adjuvant chemotherapy - Adjuvant hormone therapy - Neoadjuvant chemotherapy - Metastatic disease treatment - No active treatment *	8 (27.6%) 9 (31.0%) 1 (3.4%) 10 (34.5%) 1 (3.4%)
Employment Status - Currently employed	8 (27.6%)
Toxic Habits - Smoking history	11 (37.9%)
Education level: - Primary education - Secondary education - Higher vocational training - High School diploma - University degree	1 (3.4%) 3 (10.3%) 6 (20.7%) 5 (17.2%) 14 (48.3%)
Family History of Cancer	7 (24.1%)
Disease stage at baseline - Early-stage disease - Metastatic disease	19 (65.5%) 10 (34.5%)
Bone metastases	10 (34.5%)
Liver metastases	5 (17.2%)
Lung metastases	4 (13.8%)
Lymph node metastases	2 (6.9%)
Brain metastases	1 (3.4%)
Concomitant Medication - Antidepressants - Benzodiazepines - Statins - Antihypertensive drugs - Antidiabetic drugs	6 (20.7%) 10 (34.5%) 2 (6.9%) 4 (13.8%) 1 (3.4%)
Hypothyroidism: - No - Yes	25 (86.2%) 4 (13.8%)
Surgical Treatment - Yes - No	27 (93.1%) 2 (6.9%)
Type of Surgery - Mastectomy - Lumpectomy - No surgery	10 (34.5%) 17 (58.6%) 2 (6.9%)
Lymphadenectomy - Yes - No	8 (27.6%) 21 (72.4%)
Radiotherapy - Yes - No	19 (65.5%) 10 (34.5%)
Events During the Program - Emergency visits - Hospitalizations - Exercise-related disabling injuries	4 (13.7%) 2 (6.9%) 0 (0%)

* The patient classified as “no active treatment” had previously received neoadjuvant therapy and surgery and was assessed approximately three weeks after surgery, before initiation of subsequent adjuvant treatment.

**Table 2:** Global QoL scale. This table presents the pre- and post-intervention scores for various functional and quality of life parameters, as assessed using the EORTC QLQ-C30 and other relevant measures.

Test	PRE	PRE	POST	POST	*p*-Value (Two-Tailed)
	M	SD	M	SD	
QL2.S (Global quality of life)	58.91	18.22	70.40	13.83	**0.002**
PF2.S (Physical function)	85.06	13.76	91.95	9.15	**0.006**
RF-2-S (Role function)	74.71	24.65	89.66	13.67	**0.004**
EF-S (Emotional function)	77.01	17.35	86.78	16.14	**0.003**
CF-S (Cognitive function)	73.56	18.10	81.03	12.38	**0.034**
SF-S (Social function)	64.94	23.71	78.74	22.23	**0.005**
FA-S (Fatigue)	37.55	18.41	26.82	15.29	**0.007**
NV-S (Nausea and vomiting)	2.87	6.41	1.72	5.17	0.317
PA-S (Pain)	31.03	22.15	26.44	17.55	0.222
DY-S (Dyspnea)	16.09	19.15	9.20	15.16	0.083
SL-S (Insomnia)	47.13	30.23	39.08	30.95	0.124
AP-S (Appetite loss)	9.20	17.59	5.75	15.61	0.366
CO-S (Constipation)	17.24	24.59	13.79	20.93	0.257
DI-S (Diarrhea)	14.94	28.99	11.49	24.03	0.317
FI-S (Financial difficulties)	11.49	22.32	9.20	23.40	0.527
BMI (Body Mass Index)	25.26	4.98	25.16	4.61	0.931
Fat mass percentage	33.11	8.47	33.05	7.77	0.456
Fat mass in kg	23.61	10.88	23.43	9.72	0.855
Lean mass	44.68	5.69	44.89	5.28	0.381
Waist circumference	81.69	11.59	81.62	9.57	0.936
Hip circumference	102.00	10.97	103.35	10.66	0.053
Waist-to-hip ratio	0.80	0.07	0.79	0.05	0.336
FZA.DA (Right-hand grip strength)	20.14	4.73	20.03	4.75	0.781
FZA.IZ (Left-hand grip strength)	18.00	5.15	18.93	5.31	0.325
6-Minute Walk Test (metres)	556.21	123.60	666.38	56.075	**<0.001**

**Table 3:** Results of the Functional Movement Screen (FMS) before and after the 12-week exercise intervention. Abbreviations: PRE, pre-intervention; POST, post-intervention; DA, dominant side; IZ, non-dominant side.

Test	PRE	PRE	POST	POST	*p*-Value (Two-Tailed)
	**M**	**SD**	**M**	**SD**	
FMS.1. (Deep squat)	1.69	0.850	2.24	0.689	**0.003**
FMS.2.DA (Hurdle Step)	2.55	0.632	2.76	0.435	0.153
FMS.2.IZ (Hurdle Step)	2.59	0.682	2.83	0.384	0.100
FMS.3.DA (Incline Lunge)	2.03	0.865	2.76	0.577	**<0.001**
FMS.3.IZ (Incline Lunge)	2.10	0.860	2.76	0.577	**0.001**
FMS.4.DA (Shoulder Mobility)	2.31	1.039	2.69	0.604	0.070
FMS.4.IZ (Shoulder Mobility)	2.55	0.783	2.62	0.494	0.614
FMS.5.DA (Active Straight-Leg Raise)	2.66	0.553	2.69	0.471	0.782
FMS.5.IZ (Active Straight-Leg Raise)	2.69	0.541	2.72	0.455	0.782
FMS.6 (Trunk Stability Push-Up)	1.38	0.622	1.62	0.728	**0.008**
FMS.7.DA (Rotary Stability)	2.03	0.865	2.17	0.468	0.392
FMS.7.IZ (Rotary Stability)	2.17	0.805	2.21	0.559	0.771
Total	2.23	0.460	2.51	0.312	**0.001**

**Table 4:** Hematological and Lipid Parameters Pre- and Post-Intervention.

Biomarker	PRE (Mean)	POST (Mean)	*p*-Value
Hemoglobin (Hb)	12.80	12.97	0.348
Red cell distribution width (RDW)	15.21	14.18	0.005
Total cholesterol	195.48	188.97	0.176
Triglycerides	109.52	104.07	0.489
HDL cholesterol	60.66	64.55	0.007
LDL cholesterol	114.41	106.38	0.172

## Data Availability

The original contributions presented in this study are included in the article/Supplementary Materials. Further inquiries can be directed to the corresponding authors.

## Supplementary Materials

The following supporting information can be downloaded at https://www.mdpi.com/article/10.3390/jcm15041480/s1. Supplementary File S1—Strength and Functional Movement Assessments; Supplementary File S2—EORTC QLQ-C30 Quality of Life Assessment; Supplementary File S3—Structure of the Supervised Exercise Program.
