# Supplementary material for: Effects of a 12-Week Supervised Exercise Program on Quality of Life, Functional Capacity, and Biological Parameters in Women with Breast Cancer: A Prospective Pilot Program
Source: J Clin Med. 2026 Feb 13;15(4):1480. doi: 10.3390/jcm15041480 (PMC12942117; doi:10.3390/jcm15041480)
Supplement: Supplementary file 1 [file jcm-15-01480-s001.zip › jcm-4131572-supplementary.pdf]

Supplementary File S1 – Strength and Functional Movement Assessments

This appendix describes the assessment tools used to evaluate muscular strength and functional movement capacity in patients before and after the 12-week supervised exercise program.

A. Strength Assessments

To assess muscular strength and physical function, the following standardized tests were performed:

1. Handgrip Strength Test
   Maximal isometric handgrip strength was assessed using a calibrated hand dynamometer (Jamar® or equivalent), in accordance with guidelines from the American Society of Hand Therapists (ASHT).
   - Each participant completed two attempts per hand, with the highest value recorded.
   - The test was performed with the participant seated, shoulder adducted, elbow flexed at 90°, and forearm in neutral position.
   - The outcome was recorded in kilograms (kg) for:
     - Right hand (FZA.DA)
     - Left hand (FZA.IZ)

   Handgrip strength is a validated marker of overall muscle strength and functional status in oncologic and general populations.

2. Body Composition and Anthropometric Data
   The following measurements were collected pre- and post-intervention to provide a broader view of physical condition and muscle mass:
   - Body Mass Index (BMI)
   - Waist and hip circumference
   - Body fat mass (in % and kg)
   - Lean mass
     Measurements were obtained using bioelectrical impedance analysis (BIA), performed under standardized hydration and fasting conditions.

B. Functional Movement Screen (FMS)

The Functional Movement Screen™ (FMS) is a validated screening tool designed to detect asymmetries, movement limitations, and compensations that may increase the risk of injury or impair physical performance.

It consists of seven movement-based tests, each scored from 0 to 3:

- 0 = Pain during movement
- 1 = Inability to perform the movement correctly

- 2 = Movement performed with compensations or instability
- 3 = Movement performed correctly and without compensation

The maximum total score is 21 points.

FMS Components:

1. Deep Squat – Assesses bilateral mobility and stability of hips, knees, and ankles, as well as thoracic spine and shoulder mobility.
2. Hurdle Step – Evaluates single-leg stability and coordination during stepping motions.
3. Inline Lunge – Tests mobility and stability of the lower extremity and core under dynamic conditions.
4. Shoulder Mobility – Assesses the range of motion and symmetry of the upper extremities.
5. Active Straight-Leg Raise – Measures hamstring and calf flexibility and core stability.
6. Trunk Stability Push-Up – Evaluates core and upper-body strength and neuromuscular control.
7. Rotary Stability – Tests multiplanar trunk stability during a combined upper and lower body movement.

FMS was conducted by certified professionals following standardized protocols.

The total FMS score and individual test results were recorded before and after the 12-week intervention to evaluate changes in movement quality, balance, and functional control.

Supplementary File S2 – EORTC QLQ-C30 Quality of Life Assessment

The European Organisation for Research and Treatment of Cancer Quality of Life Questionnaire-Core 30 (EORTC QLQ-C30) is a validated, standardized instrument designed to assess health-related quality of life in cancer patients. It includes 30 items that cover multiple domains of functioning, symptom burden, and overall well-being.

Patients completed the questionnaire at baseline (pre-intervention) and at the end of the 12-week exercise program. The estimated time to complete the questionnaire was 8 to 10 minutes per patient. All participants completed the survey in a quiet environment, and assistance was provided when necessary to ensure full comprehension of the items.

Each domain or item is scored on a scale of 0 to 100. Higher scores on the Global Health Status and Functional Scalesrepresent better quality of life or functioning, whereas higher scores on Symptom Scales reflect greater symptom burden.

The table below summarizes the domains assessed in the EORTC QLQ-C30 questionnaire and their corresponding codes:

**Global health status / QoL**	**Scale**	**Functional scales**	**Scale**	**Symptom scales / items**	**Scale**
Global Health status/QoL	QL2	Physical functioning	PF2	Fatigue	FA
		Role functioning	RF2	Nausea and vomiting	NV
		Emotional functioning	EF	Pain	PA
		Cognitive functioning	CF	Dyspnoea	DY
		Social functioning	SF	Insomnia	SL
				Appetite loss	AP
				Constipation	CO
				Diarrhoea	DI

				Financial difficulties	FI

Supplementary File S3 – Structure of the Supervised Exercise Program

The supervised exercise program lasted 12 weeks and was structured as follows:

- Warm-Up: Each session began with 5–10 minutes of joint mobility and dynamic stretching exercises. The intensity was set at a Rating of Perceived Exertion (RPE) of 10–12 on the Borg Scale, corresponding to approximately 50–65% of VO_2 max according to the American College of Sports Medicine (ACSM).
- Main Aerobic Phase: This segment consisted of 10 to 30 minutes of continuous aerobic exercise performed by walking, jogging, or cycling at a moderate intensity (RPE 13–15), correlating with 75–80% of VO_2 max and 60–85% of heart rate reserve (HRR). Heart rate was monitored continuously during sessions to assess cardiovascular response.
    - Training was progressive: patients began with 10 minutes during the first two weeks. The duration was increased by 10 minutes every two weeks, reaching 30 minutes by week 6. Depending on individual tolerance and adaptation, alternation between walking and jogging was allowed (up to 85% HRR).
- Strength Training Phase: After the aerobic exercise, a general resistance training session targeting all major muscle groups was conducted using dumbbells, bars, kettlebells, and resistance bands.
    - The initial intensity was set at 10% of the estimated 1-repetition maximum (1RM). This was progressively increased by 10% every three weeks, reaching 40% of 1RM.
    - Two sets of 8 to 10 repetitions were performed for each exercise, with 2-minute rest intervals between sets.
- Cool-Down: Each session concluded with 10 minutes of static stretching to promote muscle relaxation and recovery.

All training variables (intensity, duration, density) were individually adapted based on each participant’s physical condition and treatment phase.

Supervised Resistance Training Program

**Week**	1	2	3	4	5	6	7	8	9	10	11	12

Session	1-3	4-6	7-9	10-12	13-15	16-18	19-21	22-24	25-27	28-30	31-33	34-36
**Phases**	Phase 1: Familiarization			Phase 2: Circuit Training								
**Exercises nº**	6	6	6	6	6	6	5	5	5	5	5	5
**Sets**	2	2	3	3	3	4	3	3	4	3	3	4
**Repetitions (CE intensity)**	14 (36)	14 (36)	14 (36)	12 (30)	12 (30)	12 (30)	12 (24)	12 (24)	12 (24)	8 (16)	8 (16)	8 (16)
**Estimated Intensity (%RM)**	30	30	30	40	40	40	50	50	50	60	60	60
**Total reps per exercise**	28	28	42	36	36	48	36	36	48	24	24	32
**Exercise intensity: Borg Scale (0-10)**	4-5	4-5	4-5	5-6	5-6	5-6	6-7	6-7	6-7	7-8	7-8	7-8
CE: number of repetitions actually performed out of the máximum number of repetitions that could be performed with the current load; RM: repetition maximum												

Supervised Aerobic Training Program

Week	1	2	3	4	5	6	7	8	9	10	11	12
**Session**	2	5	8	11	14	17	20	23	26	29	32	35
**Repetitions of HIIT 1 minute**				13	13	15	13	13	15	13	13	15
**Repetitions of LIT 1 minute**	20	20	25	13	13	15	13	13	15	13	13	15
**Total time (minutes)**	20	20	25	26	26	30	26	26	30	26	26	30
**Estimated Intensity (%HRR)**	50	50	60	40-70	40-80	40-80	40-85	40-85	40-85	40-85	40-85	40-85
**Exercise intensity: Borg Scale**	4-5	4-5	4-5	5-6	5-6	5-6	6-7	6-7	6-7	7-8	7-8	7-8
HIIT: high intensity interval training: LIT: low intensity training; HRR: Heart rate reserve												

Below is a session-by-session overview of the multicomponent supervised exercise program implemented over the 12-week intervention. Each session included a warm-

up, preparatory phase, main workout (aerobic and/or resistance training), and a cool-down phase with flexibility exercises. Adaptations were applied as needed based on individual limitations (e.g., neuropathies, lymphedema, PICC lines, or surgical reconstructions).

Session 1.

	**Exercise Category**	**Exercises**	**Adaptations (if needed)**
Warm-up	Aerobic Activity	5 min low-intensity aerobic activity (50%-65% HRR) walking or cycling	
	Hip, Thoracic, Shoulder Mobility<br>Sets and repetition: 2x10	Hip opener<br>Thoracic rotation in standing<br>Band pull apart	Increase ROM progressively<br>Perform unilaterally with the unaffected side.
Preparatory part	Dynamic stability exercise	Bipodal to monopodal in the frontal plane.<br>Dinamic balance: hip abd-add, hip flex-ext.<br>Movimientos dinámicos con hombros y tronco en posición monopodal	Exercises were adapted when not feasible due to neuropathy. Difficulty was progressively increased by reducing visual input.
	Core Stability exercises	Bird Dog<br>Bridge<br>Plank on knees (start from wall).<br>Crunch foot to foot.	All exercises were adapted to each participant's individual condition and clinical status. Modifications were implemented in cases of lymphedema, PICC lines, or flap-based reconstructive surgery.
Work out Resistance Training	Strength	Bilateral squat<br>Bilateral bench press<br>Lunges<br>Bilateral row<br>Bilateral swing kettlebell	Increase ROM progressively<br>Perform unilaterally with the unaffected side.
Cool-down<br>Sets and repetition: 1x10-12	Dynamic and static stretching	Hamstrings, quadriceps, pectoralis mayor, dorsal width	

Session 2.

	**Exercise Category**	**Exercises**	**Adaptations (if needed)**

Warm-up	Aerobic Activity	5-10 min low-intensity aerobic activity (50%-65% HRR) walking or cycling	
	General joint mobility for all joints (ankles, knees, hip, spine, shoulders and neck).		Perform unilaterally with the unaffected side<br>Increase ROM progressively
Preparatory Part	Core Stability exercises	Trunk rotations, tilts and flexions with a kettlebell or medicine bal.	Start from wall
Work out	Aerobic Activity	20-30 min low-intensity aerobic activity (40%-85% HRR) walking, running or cycling	
Cool-down: Sets and repetition: 1x10-12	Dynamic and static stretching	Hamstrings, quadriceps, pectoralis mayor, dorsal width	

Session 3.

	**Exercise Category**	**Exercises**	**Adaptations (if needed)**
Warm-up	Aerobic Activity	5 min low-intensity aerobic activity (50%-65% HRR) walking or cycling	
	Shoulder, Hip and ankle Mobility<br>Sets and repetition: 2x10	Squat to stand<br>Good morning<br>Pass Through	Perform unilaterally with the unaffected side<br>Increase ROM progressively
Preparatory Part	Balance and agility exercises	Single-leg static balance and dynamic balance exercises, including hip abduction–adduction and flexion–extension movements	If not feasible due to neuropathy, exercises were adapted. Difficulty was progressively increased by reducing visual input.
	Dinamic Core Stability exercises	Bird Dog<br>Bridge	Start from wall

		Plank on knees.<br>Crunch foot to foot. Si no puede, se hace de pie en plano frontal.	
Resistance Training	Strength	Bilateral deadlift<br>Bilateral row<br>Lunges<br>Bilateral bench press<br>Bilateral squat	Increase ROM progressively<br><br>Perform unilaterally with the unaffected side
Cool-down: Sets and repetition: 1x10-12	Dynamic and static stretching	Hamstrings, quadriceps, pectoralis mayor, dorsal width	
